# Porcine Dendritic Cells and Viruses: An Update

**DOI:** 10.3390/v11050445

**Published:** 2019-05-16

**Authors:** Giulia Franzoni, Simon P. Graham, Silvia Dei Giudici, Annalisa Oggiano

**Affiliations:** 1 Istituto Zooprofilattico Sperimentale della Sardegna, 07100 Sassari, Italy; silvia.deigiudici@izs-sardegna.it (S.D.G.); annalisa.oggiano@izs-sardegna.it (A.O.); 2 The Pirbright Institute, Ash Road, Pirbright GU24 0NF, UK; simon.graham@pirbright.ac.uk; 3 School of Veterinary Medicine, University of Surrey, Guildford GU2 7AL, UK

**Keywords:** pig, dendritic cells, ASFV, CSFV, PCV2

## Abstract

Several viral infections of swine are responsible for major economic losses and represent a threat to the swine industry worldwide. New tools are needed to prevent and control endemic, emerging, and re-emerging viral diseases. Dendritic cells (DC) play a central role in linking the innate and adaptive arms of the immune system, so knowledge regarding their interaction with pathogens is necessary to understand the mechanisms underlying diseases pathogenesis and protection. In the first part of this review, we provide an update on the heterogeneous cell subsets that comprise the porcine DC family. In the second part of this review, we provide an overview of how three viruses, affecting pork production at a global level, African swine fever virus (ASFV), classical swine fever virus (CSFV), and porcine circovirus 2 (PCV2), modulate DC function.

## 1. Introduction 

Pork production accounts for more than one-third of all meat production worldwide, and infectious diseases are its primary constraint. Globalisation and industrialisation of the pig industry has contributed to the emergence and spread of pathogens, including viruses [1]. A recent study, which used published research as an indicator of research priorities, revealed that the most important swine viruses worldwide between 2006–2016 were: swine influenza virus (SwIV), pseudorabies virus (PRV), foot-and-mouth disease virus (FMDV), porcine reproductive and respiratory syndrome virus (PRRSV), classical swine fever virus (CSFV), African swine fever virus (ASFV), and porcine circovirus 2 (PCV2) [1]. The top published virus worldwide was the zoonotic SwIV, and four of the other top published viruses (PRV, FMDV, ASFV, and CSFV) cause diseases listed as notifiable by the OIE World Organization for Animal Health. PRRSV and PCV2 are not notifiable pathogens, as they are endemic in the majority of pig producing countries and they compromise pork production by their effect on mortality, reproduction, and growth [1]. New tools are needed to aid in preventing the spread of devastating epizootic viruses and reducing the incidence of the endemic ones. Vaccines play a critical role in preventing, managing, and controlling viral diseases. In order to rationally design vaccines, it is mandatory to acquire information regarding both humoral and cellular immune responses against pathogens [2]. Dendritic cells (DC) are the most potent antigen-presenting cells (APCs) and, as such, link the innate and adaptive arms of the immune system [3]. A better understanding of how these cells interact with pathogens is of major importance to comprehend both diseases pathogenesis and mechanisms behind immunological protection, generating important information that underpins vaccine research and development efforts. Therefore, in the first part of this article, we provide an update on our understanding of porcine DC, which has benefitted from a number of recent studies that phenotypically and functionally characterised both blood-borne and tissue-resident DC subsets. Since DC interactions with SwIV, PRRSV, and FMDV were recently reviewed in detail [4,5], we discusse, in the second part of this review, DC interaction with three other viruses that threaten the swine industry: ASFV, CSFV, and PCV2.

## 2. Dendritic Cells 

### 2.1. Dendritic Cells: A Heterogeneous Family

DC are a class of bone marrow-derived cells that were originally identified by Steinman and Cohn in the early 1970s [6]. They are regarded as professional APCs and are considered to be the sentinels of the immune system [3]. To execute their role as sentinels, they are equipped with several pattern recognition receptor (PRRs), which recognize pathogen-associated molecular patterns (PAMPs) and can detect foreign antigen at the entry sites, such as skin and mucosae [7]. They can uptake and process proteins, and then migrate into the secondary lymphoid tissues to present the processed antigens to lymphocytes [3]. In most tissues, DC reside in an immature state. Immature DC lack co-stimulatory molecules that are required for naïve T cells activation, such as CD40, CD54, and CD80/86, and the majority of major histocompatibility complex (MHC) class II molecules are located within the intracellular compartment [8]. On the contrary, mature DC are characterized by reduced endocytic/phagocytic activity and, instead, present accessory molecules and the extracellular expression of MHC class II [8,9]. DC present the unique ability of priming naïve T lymphocytes and possess a key role in controlling T lymphocyte activation and regulation [8]. DC also have important roles in B-cell activation and the regulation of antibody synthesis, producing factors that are important for their proliferation, differentiation, and isotype switching [8]. They can also directly interact with B cells, directly transferring antigens to naïve B cells to initiate specific antibody synthesis [10]. In addition, DC are involved in natural killer (NK) cell activation by releasing both IL-12, which increases NK cytotoxicity, and IL-15, which has a critical role in NK cell survival, expansion, and function [11,12]. The cross-talk between NK cells and DCs is bidirectional, and activated NK cells can further mature DC, which enhances their ability to stimulate T cell responses [13]. DC also play a role in γδ-T cell activation, through the release of pro-inflammatory cytokines and expression of ligands for γδ TcR and natural killer receptors (NKRs) [14]. 

DCs are a heterogeneous family, being broadly subdivided in three major subsets: conventional DC1 (cDC1), conventional DC2 (cDC2), and plasmacytoid DC (pDC) [15]. As recently reviewed by Collin and Bigley (2018), cDC1 present high capacity to promote CD4^+^ T helper 1 (TH1) and NK responses, through IL-12 secretion, and to cross-present exogenous antigen via MHC class I to CD8^+^ T cells, whereas cDC2 promote CD4^+^ T helper 2 (TH2) and 17 (TH17) responses [15]. In contrast to mice, in humans, cross-presentation to CD8^+^ T cells is performed by both cDC1 and cDC2 [15]. pDC were first identified in human as CD4^+^CD3^−^ cells with a plasma cell-like morphology and they were initially erroneously considered to be cells of the lymphoid lineage [16]. These cells selectively express Toll-like receptor TLR-7 and TLR-9 and they are specialized in secreting large amounts of type I IFN following viral stimulation [17].

The heterogeneity of DCs from different organs is also remarkable. In humans, Granot et al. (2017) observed that cDC subsets from mucosal sites (lung and intestines) presented different composition and higher cDC2/cDC1 ratios when compared to lymph nodes and demonstrated that cDC subsets distribution was mainly a function of the specific tissue [18]. The authors observed that cDC2 presented higher maturation and migration properties as compared to cDC1, and mature cDC2 in mucosal-draining lymph nodes expressed tissue-specific markers that were derived from their original mucosal site [18]. These results revealed tissue-specific DC maturation, associated with migration phenotypes [18].

Summerfield and McCullough firstly reviewed porcine DC in 2009 [19]. Each species has its own peculiarities, so, in this article, we provide an update on the phenotype and functions of porcine DC subsets in the blood and different organs. 

### 2.2. Porcine Blood DC

**Figure 1:**
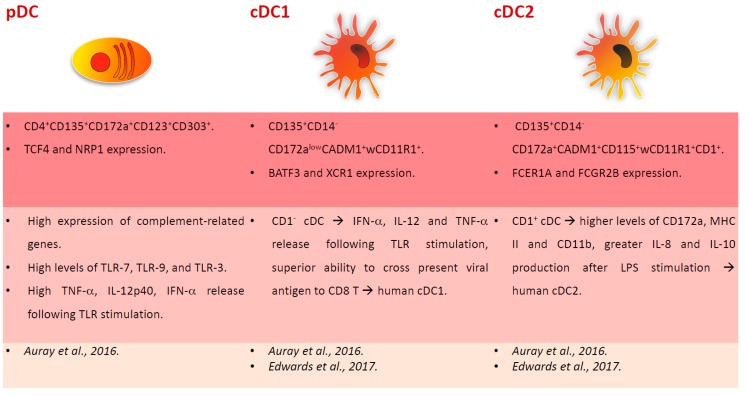
Major subsets of dendritic cells (DC) in pig blood. Phenotypical and functional descriptions of pig blood DC subsets (pDC, cDC1, cDC2), with the corresponding references at the bottom of the figures.

In 2003, Summerfield et al. provided the first characterization of blood DC subsets and identified CD172a^+^CD14^−^CD4^−^ cells that resembled human cDC, and CD172a^+^CD14^−^CD4^+^ cells orthologous to human pDC, which represented 0.2–0.6% and 0.1–0.3% of peripheral blood mononuclear cells (PBMC), respectively [20]. Porcine blood cDC presented an MHC II^+^CD80/86^+^CD1^+/−^CD4^−^CD14^−^ phenotype and up-regulated both MHC II and CD80/86 after a short culture period (24 hours) with granulocyte-macrophage colony-stimulating factor (GM-CSF) and interleukin (IL)-3. Blood pDC instead presented a CD4^+^MHC II^low^CD80/86^low^CD1-CD8^−/low^CD16^−/low^CD45RA^−/low^ phenotype and had a high IL-3 binding capacity. This cytokine promoted pDC survival in vitro [20]. As expected, pDC produced high levels of type I IFN after stimulation with transmissible gastroenteritis virus (TGEV). Both freshly isolated DC subsets presented high endocytic activity, which decreased after in vitro culture [20]. In 2016, Summerfield’s group provided a further characterization of blood DC subsets, through multicolour flow cytometry, cell sorting, and RNA sequencing [21]. pDC presented a CD4^+^CD135^+^CD172a^+^CD123^+^CD303^+^ phenotype and selectively expressed TCF4 and NRP1. Unlike in humans and mice, porcine pDC presented a high expression of complement-related genes (C2, C3, C5, and CD93), which suggests a prominent role of these cells in complement biology when compared to other species. As expected, porcine pDC expressed the highest levels of TLR-7 and TLR-9 expression, but, unlike human and mice, this subset also expressed the highest levels of TLR-3 [21]. The porcine equivalent of cDC1 and cDC2 were identified and characterized: cDC1 were CD135^+^CD14^−^CD172a^low^CADM1^+^wCD11R1^+^ and expressed BATF3 and XCR1, whereas cDC2 presented a CD135^+^CD14^−^CD172a^+^CADM1^+^CD115^+^wCD11R1^+^CD1^+^ phenotype and expressed FCER1A and FCGR2B [21]. In pigs, cDC1 presented relatively high levels of TLR-9, which resembled mice but not human cDC1 [21]. The responses to TLR ligands were also assessed and porcine pDC were the most important source of TNF-α, IL-12p40, and IFN-α. In pigs, it appears that pDC are an important source of IL-12, whereas both cDC subsets were unable to produce this cytokine following in vitro stimulation. TLR stimulation upregulated the surface expression of MHC II and co-stimulatory molecules on cDC, and this was enhanced by the presence of pDC, especially for TLR-7 and TLR-9 ligands [21]. A recent study also focused on porcine blood cDC characterization, which were segregated in two different subsets by their expression of CD1 [22]. The authors observed that CD1^−^ cDC secreted IFN-α, IL-12, and TNF-α following TLR stimulation and presented a superior ability to cross-present viral antigen to CD8 T cells, thus resembling human cDC1. On the contrary, CD1^+^ cDC, which expressed higher levels of CD172a, MHC II, and CD11b when compared to the other cDC subset, produced greater amounts of IL-8 and IL-10 after LPS stimulation, suggesting that they resemble more human cDC2, involved in promoting a TH2 response [22]. Figure 1 summarizes the phenotypical and functional descriptions of pig blood DC subsets.

The blood DC subsets in newborn piglets were also recently studied. It was reported that neonatal pigs (four-day-old) had similar DC subset proportions within the total PBMC population as compared to immunocompetent older (12-week-old) weaned piglets, whereas the neonates presented a higher proportion of monocytes [23]. Neonatal cDC1 expressed higher levels of MHC II, instead no differences in CD80/86 levels on DC subsets were detected between the new-born and older piglets [23]. Pam3Cys (TLR-2 ligand) induced a stronger TNF-α expression in pDC and a higher CD80/86 up-regulation in cDC1 in new-borns when compared to older piglets. Furthermore, CpG oligodeoxynucleotide (ODN) (TLR-9 ligand) induced a higher IL-12p40 expression in neonatal pDC. Those differences were not linked to variation in TLR-2 and TLR-9 expression, which were similar between the neonatal and older piglet CD3-depleted PBMC at steady-state [23]. In another study, the responses of neonatal blood derived DC to poly(I:C) (TLR-3 ligand), LPS (TLR-4 ligand), and imiquimod (TLR-7 ligand) were assessed [24]. Neonatal blood DC showed significant increases in CD80/86 expression that was similar to blood DC that was isolated from older (eight-week-old) piglets. TLR-7 stimulation induced significantly higher expression of several chemokines (CCL2, CCL3, CCL10, and CXCL8) by neonatal blood DC when compared to their older piglet counterparts. In addition, the stimulation of these cells with TLR-7 ligand induced higher levels of IL-12 as compared to older animals. Interestingly, despite equal proportions of pDC, neonatal but not older piglet blood DC produced IFN-α after TLR7 or TLR3 ligand stimulation. In addition, neonatal blood DC displayed a greater ability to induce lymphocyte proliferation in the mixed leucocyte reaction (MLR) assay [24]. These studies suggest that TLR-2, TLR-3, TLR-7, and TLR-9 ligands could be promising candidates for neonatal adjuvant application, although further studies on DC isolated from neonatal lymphoid tissues are required [23,24].

### 2.3. Monocyte-Derived DC 

Due to the low frequency of DC in blood and tissues, cDC can be generated in vitro by culturing monocytes in medium that is supplemented with recombinant GM-CSF and IL-4 [25,26]. These monocyte-derived DCs (MoDC) present a dendritic morphology, a MHCII^high^CD80/86^+^CD172a^+^CD1^+^ phenotype, and a strong T-cell stimulatory capacity [25,26]. Interestingly, CD163^+^ monocytes presented a higher ability to differentiate to moDC (higher levels of MHC II and CD80/86 and more efficient in promoting lymphoproliferation) as compared to CD163^−^ monocytes [27]. The differentiation of porcine MoDC may also be achieved through the use of IL-13 instead of IL-4 [28]. 

moDC generated through IL-4/IL-13 and GM-CSF incubation are in an immature state and present a high endocytic activity and lower expression of MHC II and co-stimulatory molecules when compared to their mature counterparts [8,19]. The maturation of porcine moDC can be achieved through IFN-α and TNF-α stimulation, which results in increased MHC and CD80/86 expression [29]. Other maturation stimuli have been tested; for example, Singleton et al. (2016) matured moDC through 24 hours incubation with LPS, IFN-γ, TNF-α, IL-6, IL-1β, and prostaglandin E. Maturation with this cocktail resulted in no morphological changes, although moDC were less adherent and presented a higher expression of CD80/86 and CD83 and lower levels of CD14 [30]. 

A comparison between moDC (generated through incubation with IL-4 and GM-CSF) and blood DC (a mixed pDC-cDC population) was performed [31]. Both of the populations were able to uptake foreign antigens, but blood DC activity was lower than moDC. In response to LPS stimulation, both populations upregulated CD80/86 and increased CCR7 gene expression. Both cell types stimulated primed T-cell proliferation to the same extent, whereas moDC induced a stronger proliferation of naïve T cells [31]. 

### 2.4. Porcine Skin DC

The skin provides an effective barrier against wide range of pathogens and environmental challenges, and the network of skin-associated immune cells, also called the skin immune system (SIS), ensures the protective function of this organ [32]. The immunology of this organ in pigs was reviewed in 2015 [32]. Swine skin is regarded as the best structural model for human skin and it has been used to test new vaccination devices [33,34]. It is essential to characterise the skin-resident immune populations in this species, especially resident DC, in order to understand its response to invading pathogens and to predict the outcome of intradermal vaccination. 

In the past, porcine skin DC were segregated in four subpopulations: Langerhans cells (LC), CD172a^−^ dermal DC, CD163^+^ dermal DC, and CD163^low^ dermal DC [35]. LC reside in the basal epidermis (the avascular superficial layer of the skin) and, unlike the other DC subsets, they originate from embryonic precursors, mainly foetal liver monocytes, which are recruited to the epidermis during embryonic development. They are capable of local self-renewal, but, in case of severe inflammation, LC can be generated from monocytes and other uncharacterised myeloid precursors [15,32]. LC are involved in maintaining epidermal health and tolerance to commensal microorganisms; nevertheless, they are able to respond to viruses and other intracellular pathogens [15]. Porcine LC express langerin (CD207) and represent more than 50% of skin DC [36]. They were also described as CD172a^+^CD163^−^CD16^−^CADM1^+^CD207^+^MHCII^+^ [35].

**Figure 2:**
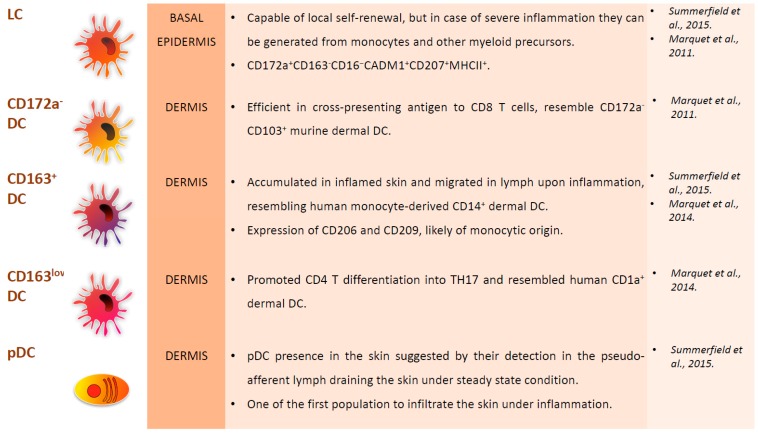
Major subsets of DC in pig skin. Location, phenotypical and functional descriptions of pig skin DC subsets, with the corresponding references at the right of the figures.

Three DC subsets have been observed in the dermis, being discriminated by their expression of CD172a and CD163. All of these subsets were also detected in skin-draining pseudo-afferent lymph, which suggests that they are able to migrate from the skin to local lymph nodes [35]. CD172a^−^ dermal DC are efficient in cross-presenting antigen to CD8^+^ T cells and they resemble CD172a^−^CD103^+^ murine dermal cDC1 [35]. CD172a^+^CD163^low^ dermal DC instead resemble human CD1a^+^ dermal cDC2 and promote CD4^+^ T differentiation into TH17 [34]. CD172^+^CD163^+^ dermal DC are accumulated in inflamed skin and they migrate in lymph upon inflammation, resembling human CD14^+^monocyte-derived dermal DC [34]. CD172^+^CD163^+^ cells also expressed CD206 and CD209 and another study reported that CD209^+^ DC in the dermis are likely of monocytic origin, like in human skin [32]. The presence of pDC in the skin was never directly described, but they were detected in the pseudo-afferent lymph that drains the skin under steady-state conditions, which suggests their presence in the skin. pDC are also one of the first populations to infiltrate the skin during inflammation [32]. Figure 2 summarizes the phenotypical and functional descriptions of porcine skin DC subsets.

### 2.5. Porcine DC in Tonsils and Lymph Nodes

**Figure 3:**
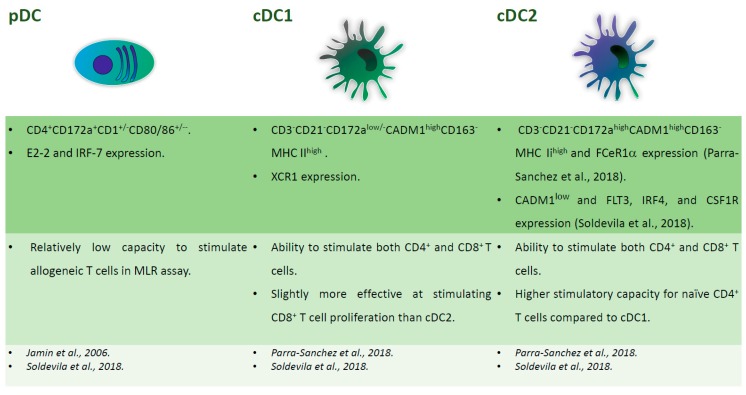
Major subsets of tonsil DC. Phenotypical and functional descriptions of pig tonsil DC subsets (pDC, cDC1, cDC2), with the corresponding references at the bottom of the figures.

In 2006, Jamin and colleagues first characterised porcine DCs in these organs. They defined two populations: CD172a^+^CD11R1^+^CD1^+/−^CD80/86^+/−^ and CD172a^+^CD4^+^CD1^+/−^CD80/86^+/−^ DCs, which correspond to cDC and pDC, respectively [37]. In 2018, Soldevila et al. characterised five myeloid cells population resident in porcine tonsils, of which three were DC subsets. pDC were CD4^+^ and were defined by expression of E2-2 and IRF-7 markers, cDC1 expressed CADM1^high^CD172a^low^ and high levels of XCR1, whereas cDC2 were CADM1^low^ and expressed FLT3, IRF4, and CSF1R [38]. pDC presented relatively low capacity for stimulating allogeneic T cells in an MLR assay, whereas the two cDC subsets were able to stimulate both the CD4^+^ and CD8^+^ T cells, with little differences between the subsets. cDC2 showed the highest stimulatory capacity for naïve CD4^+^ T cells and cDC1 were slightly more effective in stimulating CD8^+^ T cell proliferation. The authors speculated that the similar capacity of the two cDC subsets to activate CD8 T cells might be advantageous in the tonsils, being one of the major sites of pathogen entry [38]. Another study characterising the secondary lymphoid tissue-derived DC identified cDC as CD3^−^CD21^−^CD163^−^MHC II^high^CADM1^high^ cells, further divided in two subsets that are based on CD172 expression. cDC1, with low/no levels of CD172a, also expressed XCR1, whereas, CD172a^high^ cDC2 expressed FCeR1α [39]. Like in humans, DEC205, a receptor that promotes antigen presentation, was differentially expressed on porcine tonsil and lymphoid tissue cDC independently of cDC1 and 2 subsets. This is in contrast with blood, where all cDC expressed this marker [21,39]. Figure 3 summarizes the phenotypical and functional descriptions of porcine tonsil DC.

### 2.6. Porcine Respiratory DC

**Figure 4:**
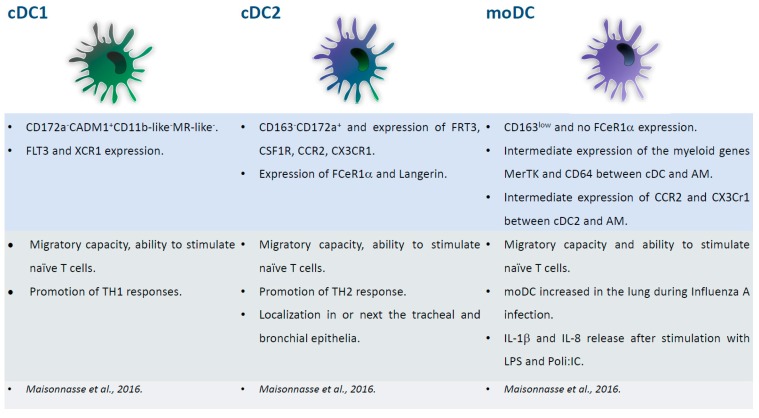
Major subsets of respiratory DC. Phenotypical and functional descriptions of pig lung DC subsets (cDC1, cDC2, moDC), with the corresponding references at the bottom of the figures.

Three DC subsets have been identified in the porcine lung: cDC1, cDC2, and inflammatory moDC [40]. All of the subsets presented migratory capacity and the ability to stimulate naïve T cells. cDC1 stimulated TH1 responses, whereas cDC2 promoted TH2 response. The latter subset expressed FCeR1α and langerin and were localized in or next to the tracheal and bronchial epithelia [40]. FCeR1α was absent in moDC, which presented with low levels of CD163. moDC increased in the lung during influenza A virus infection, and secreted IL-1β and IL-8 when stimulated with LPS and poly(I:C), suggesting their pathological role during influenza [40]. The study of lung DC is challenging, and thus in several species bronchoalveolar lavage (BAL), which is an easy non-traumatic procedure, is used to study myeloid populations’ resident in the respiratory tract. A comparison between BAL macrophages/DC and their interstitial counterpart in pigs was recently performed [41]. As expected, there was a prevalence of alveolar macrophages in the BAL, but rare DC were identified and they were highly similar to their interstitial counterpart. The results validated the study of BAL DC as a surrogate of their parenchymal counterpart in pigs [41]. Interestingly, differences between respiratory DC in two pig breeds (Pietrain and Duroc) were observed. Lung DC were infected in vitro with PRRSV and their responses to infection were investigated using RNA-sequencing. The virus replicated differently in lung DCs from the two breeds and Duroc lung DCs reacted more strongly to PRRSV infection. The data suggested that this more efficient response was linked to IL-1β up-regulation. Overall, the data regarding DC-virus interaction underlined the important role of genetics in pig responses to PRRSV [42]. Figure 4 summarizes the phenotypical and functional descriptions of pig respiratory DC subsets.

## 3. Dendritic Cells and Viruses 

### 3.1. DC and ASFV

**Figure 5:**
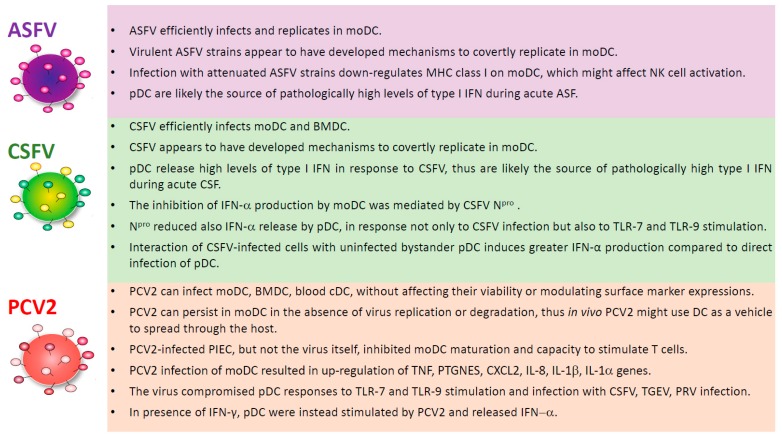
Interaction of DC with African swine fever virus (ASFV), classical swine fever virus (CSFV), and porcine circovirus 2 (PCV2). Modulation of DC subsets phenotype/functionality by ASFV (top), CSFV (middle), PCV2 (bottom).

African swine fever (ASF) is a contagious viral disease of domestic pigs and wild boars. Its aetiological agent is the African swine fever virus (ASFV), a large, enveloped DNA virus [43]; virulent isolates cause acute haemorrhagic fever in domestic pigs, which usually die in less than two weeks post-infection [44]. There is no licensed vaccine or treatment available, and thus ASF is currently considered to be one of the most devastating viral disease of domestic pigs, currently being present in many sub-Saharan African countries, Russian Federation, Trans-Caucasus, Europe, and Southeast Asia [45,46]. ASFV infects the immune cells of the myeloid lineage, with monocytes and macrophages being considered its primary target cells [47]. As we recently reviewed, few studies have analysed ASFV-DC interactions [48]. Golding et al. (2016) described that porcine PBMC enriched for DC (by depletion of CD3^+^ T cells, CD14^+^ monocytes and CD21^+^ B cells) released high levels of type I interferon in response to ASFV infection, which suggests that pDC might be the source of type I interferon in animals undergoing acute ASF [49]. Nevertheless, using a mixed DC population, the authors could not exclude that cDC released type I IFN following infection [49]. Instead, our group conducted an in vitro study that was focussed on the interaction of porcine moDC with ASFV strains of diverse virulence [50]. All of the tested ASFV strains (BA71V, NH/P68, 22653/14) infected moDC, which supported productive virus replication, although the culture-adapted BA71V strain replicated less efficiently than the other two strains. Infection with attenuated (BA71V and NH/P68) but not virulent (22653/14) ASFV resulted in MHC I down-regulation on moDC [50], which, in vivo, might affect NK cell activation [51], which has been positively correlated to survival after ASFV infection [52]. None of the ASFV strains tested induced a strong cytokine response from immature or IFN-α/TNF-α matured moDC: no detectable release of IFN-β, IL-1β, IL-6, IL-8, IL-10, IL-12, IL-18, and TNF-α was observed following ASFV infection [50]. In addition, it was recently observed that moDC infection with ASFV strains of diverse virulence resulted in a decreased phagocytic ability of these cells [53]. Overall, these results suggest that virulent ASFV isolates have evolved mechanisms to support covert replication in cDC. More studies that are focused on ASFV-blood DC interactions are needed, which take into consideration the heterogeneity within this family. In addition, studies that are focused on ASFV interaction with DC from various tissues are required. In the past, it was reported that ASFV can infect skin-derived DC [54] and interdigitating DC in mandibular lymph nodes [55], but in the last decades, many steps forward have been taken in the characterisation of DC in different organs. Therefore, future studies on ASFV modulation of DC phenotype and function in skin, tonsils, lymph nodes, and other organs should consider the new knowledge of the DC ‘system’ and take advantage of an improved toolbox for the analysis of the porcine immune system. Figure 5 summarizes the interactions of ASFV with different DC subsets.

### 3.2. DC and CSFV 

Classical swine fever (CSF) is a contagious and often fatal disease of domestic pigs and wild boar and, as mentioned before, it is classified as a notifiable disease by the OIE [56]. In the last ten years, CSF outbreaks were detected in Eastern Europe, South East Asia, Japan, Latin America, and Russia [46]. The etiological agent is the classical swine fever virus (CSFV), a small (40–60 nm in diameter) enveloped positive stranded RNA virus, and a member of the *Pestivirus* genus within the *Flaviviridae* family [57]. The virus has four structural proteins (core, E^rns^, E1, and E2) and eight non-structural proteins (N^pro^, p7, NS2, NS3, NS4A, NS4B, NS5A, and NS5B) [57]. Endothelial cells, leukocytes, and some epithelial cells, like the tonsillar crypt epithelial cells, are its major target cells in vivo [56]. In endemic regions the disease is mainly controlled by prophylactic vaccination with live attenuated vaccines, such as C-strain, which confer an effective, rapid, and solid immune protection. Nevertheless, these live attenuated vaccines lack DIVA (differentiating infecting and vaccinated animals) capability, thus are not used in the European Union (EU) [58,59]. In the EU, the current eradication program is based on strict stamping out strategy with restriction of movements of pig and pig products. In the light of the huge economic damaged that is caused by CSF outbreaks, significant efforts have been made towards the development of CSF marker vaccines [59]. In 2014, the CP7_E2alf, a chimeric virus with a bovine virus diarrhoea virus-1 (BVDV-1) backbone containing the CSFV envelope protein, E2, was licenced by the European Medicines Agency [59]. CSFV and BVDV-1 are considered in two different species, presenting different host range and more than 25% differences at the genome sequence level [60]. This chimeric vaccine is innocuous and induces immunity in wild boar after oral immunization, thus it might replace live attenuated CSF vaccines in wild boar immunisation [59].

Several studies have focused on CSFV-DC interactions in order to gain insight into the immunomodulatory characteristics of this virus. CSFV is highly efficient in infecting and replicating in either moDC or bone marrow-derived DC (BMDC), although this infection did not result in any morphological, phenotypical (MHC I, MHC II, CD80/86 expression), or functional modulation of these cells [29]. In addition, CSFV did not interfere with DC maturation (using IFN-α and TNF-α) or its ability to present antigen and stimulate T cell responses [29]. The virus was unable to induce any cytokine response by these cells and it did not interfere with TNF-α or IL-6 induced by LPS or poly(I:C), but affected IFN-α response to poly(I:C) stimulation [29]. These results suggested that CSFV has developed mechanisms to prevent antiviral responses and to covertly replicate in DC [29]. The inhibition of IFN-α production by CSFV-cells was observed in other cell types and it is linked to the blocking of IRF3 by the CSFV protein N^pro^ [61,62]. The role of N^pro^ on moDC modulation was further investigated: Bauhofer et al. (2005) observed that N^pro^ deleted mutants, but not wild-type CSFV, induced maturation (CD80/86 upregulation) and type I IFN production by moDC. N^pro^ deletion did not influence the dsRNA levels during CSFV infection, which suggests that this protein regulates type I IFN induction downstream of dsRNA detection [63]. In addition, it was reported that CSFV N^pro^ prevented type I IFN sensitisation of BMDC [64]. The role of nuclear factor kappa B (NF-κB) during CSFV infection was also investigated [65]. NF-κB is a transcription factor, which plays a key role in initiating the responses to pathogens [66]. CSFV infection did not result in p65/RelA translocation from the cytoplasm to the nucleus and it did not alter the expression of p65/RelA and IkBα, which indicates that NF-κB remained inactive during moDC infection [65]. These results suggested that CSFV controlled the activation of this transcription factor, inhibiting its antiviral effect and thus evading the host immune responses [65]. 

Several authors also assessed the interaction of CSFV with cDC and pDC. Jamin et al. (2008) analysed the activation of both cDC (CD172a^+^CD11R1^+^) and pDC (CD172a^+^CD4^+^) at early time points after CSFV infection [67]. Both subsets, in both blood and lymphoid organs, were susceptible to CSFV infection, which resulted in their maturation and activation, as demonstrated by CD1a down-regulation, CD80/86 upregulation, and cytokine expression [67]. cDC expressed TNF-α and IL-10, whereas, pDC mainly expressed IFN-α and IL-12. The tonsils and blood pDC also expressed TNF-α. Pathologically high levels of IFN-α were detected in serum early post-infection, and CSFV-infected pDC were probably the source [67]. A previous study reported that pDC release IFN-α after CSFV infection, with higher responses by pDC from immunised when compared to naïve pigs [68]. CSFV N^pro^ can block type I IFN production by cDC more efficiently than pDC; IRF7, which is more prominent in pDC, is less affected by this protein [69]. Nevertheless, CSFV N^pro^ negatively regulates IRF7, probably through interference with its turnover, reducing IFN-α release by pDC, not only after CSFV infection, but also in response to other stimuli [69]. Moreover, it has been recently shown that interaction of CSFV-infected cells with uninfected bystander pDC induces greater IFN-α production when compared to direct infection of pDC [70]. Viral RNA transfer dependant on cell contact between pDC and infected cells; intact cytoskeleton and lipid rafts of the donor cells were required for this effect. The ability to sense virus-infected cells is advantageous for defence against viruses that are mainly cell-associated, like pestiviruses. Interestingly, the RNase activity of CSFV E^rns^ limited this pathway of pDC activation, which represents a survival strategy for the virus [70]. Type I IFN release by CSFV-infected pDC was linked to the partial activation of NK and γδ-T cells [71]. We observed that pDC release high levels of IFN-α in response to both virulent and attenuated CSFV strains, and this cytokine led to MHC II up-regulation on NK and γδ-T cells. In addition, a similar NK/γδ-T cell phenotype was most strikingly observed on cells that were isolated from tonsils and retropharyngeal lymph nodes during infection with virulent CSFV. This effect inversely correlated with protection and it was not associated with the ability of NK and γδ-T cells to release IFN-γ or to express perforin, which would instead have been effective against CSFV [71]. We speculated that exacerbated type I IFN may compromise the development of a protective cellular immune response through the induction of MHC-II up-regulation on NK/γδ-T cells, thus impairing the professional DC-induced T cell responses in lymphoid tissues due to competitive antigen-presentation [71]. The exacerbated pDC-mediated type I IFN response against CSFV is likely to also be related to the induction of lymphocyte apoptosis, which further antagonizes the development of a protective T cell response. It was observed that virulent CSFV strains induced pathologically high levels of systemic IFN-α, which were detectable from two days post-infection [72,73]. This type I IFN response was correlated to the virulence of the viral strain used, with attenuated strains not inducing any detectable serum level of this cytokine. Lymphocyte depletion during infection with virulent CSFV strains was attributed to induction of apoptosis in uninfected bystander lymphocytes, where the up-regulation of apoptosis-related IFN stimulated genes (ISG) was described [74,75]. Tumour necrosis factor-related apoptosis-inducing ligand (TRAIL) released by CSFV-infected cells might also cause CSFV induced lymphopenia [76]. It can be speculated that the lower replication of live attenuated CSFV in target cells, like DC, might result in lower release of IFN-α/TRAIL and other pro-apoptotic molecules when compared to virulent isolates, “buying time” to develop a protective adaptive immune response. Figure 5 summarizes interactions of CSFV with different DC subsets.

### 3.3. DC and PCV2

PCV2 is a small non-enveloped DNA virus linked with several syndromes, commonly known as porcine circovirus–associated diseases (PCVAD), which compromises pork production by its effect on piglet mortality, reproduction, and growth [1,77]. Infection with this virus can be subclinical or associated with different pathological features, like PCV2 systemic disease (PCV2-SD), lung disease (PCV2-LD), enteric disease (PCV2-EC), reproductive disease (PCV2-RD), and porcine dermatitis and nephropathy syndrome (PDNS) [77]. How PCV2 causes such diseases is still largely unknown and a combination of different elements (virus, host, and environment) likely determine whether the infection results in subclinical disease or fatal outcome [78,79]. PCV2 is widespread worldwide in pig and wild boar populations, with a high circulation rate [1,80]. PCV2 is a member of the genus *Circovirus*, within the family *Circoviridae*, has a single-stranded circular genome of 1766–1768 nucleotides. During its replication, a dsDNA intermediate is generated, which encodes genes in both the viral (ORF1, also called Rep gene) and the complementary (ORF2, also called Cap gene, and ORF3) strands [79]. This virus presents the highest genetic variability among single-stranded-DNA viruses and it is characterised by a high rate of nucleotide substitution, being close to that of RNA viruses [81]. Currently four genotypes (PCV2a, PCV2b, PCV2c, and PCV2d) have been identified [79]. 

This virus deeply affects the functionality of the pig immune system, with massive lymphoid depletion being observed in PCV2-SD, whereas asymptomatic PCV2-infected pigs are able to mount humoral and cellular immune responses, clearing or limiting the infection. Mechanisms beyond PCV2 immunopathogenesis are still poorly understood and many knowledge gaps need to be filled [78,79,82]. In addition, the available vaccines do not induce sterile immunity and PCV2 continues circulating in farms applying vaccination [82]. A better comprehension on PCV2 interaction with the host immune system is required to prevent and control PCVAD.

PCV2 interaction with DC was reviewed in 2009, 2010, and 2012 [78,83,84], and in this article we will provide an update. In 2003, Vincent et al. observed that PCV2 infected BMDC and moDC *in vitro*, without effects on their viability or the modulation of surface marker expression (MHC I, MHC II, CD80/86, CD14, CD16, CD25). However, the virus was unable to replicate in these cells, even when they were co-cultured with syngeneic T cells, although PCV2 infectivity remained intact throughout the experiment (five days) [85]. Steiner et al., (2008) observed that moDC were unable to support PCV2 replication, despite their efficient ability in virus binding and uptake. On the contrary, all of the other cell lines that were tested in the study (endothelial cell line PEDSV.15, aortic endothelial cell, gut epithelial cell, fibrocyte) supported detectable virus replication [86]. PCV2 can persist in DC in the absence of virus replication or degradation, thus it can be speculated that these cells could serve as a vehicle for virus that is spread through the host [85,86]. Even if the virus did not directly modulate moDC function, their function was impaired by co-culture with PCV-2 infected porcine iliac endothelial cells (PIECs). PCV2-infected PIEC, but not the virus itself, inhibited moDC maturation (assessed by MHC II and CD80/86 expression) and weakened their ability to stimulate allogeneic T cells, which suggests that PCV2 infection of endothelial cells might represent a pathogenic mechanism of this virus [87]. In contrast, Kekarainen et al. observed that PCV2 induced both IL-10 and IL-12 release by BMDC, and IL-12 secretion was triggered also by PCV2 virus-like particles [88,89]. In addition, PCV-2 inhibited IFN-α release by BMDC in response to PRV infection, and a similar immunomodulatory effect was induced by PCV2-derived CpG sequences, which were mainly located within the Rep gene [88,89]. Another study reported that moDC strongly responded to the PCV-2 infection, with increased expression of 40 genes, mainly immune-related and pro-apoptotic [90]. Using both microarray and RT-PCR, the authors observed that PCV2 infection up-regulated TNF, PTGNES, CXCL2, IL-8, IL-1β, and IL-1α, which might be related to the lymphocyte depletion and inflammation observed in PCVAD [90]. Lin et al. studied which factors facilitated PCV2 replication in DC [91]. The virus was unable to replicate in either untreated or LPS-treated moDC, and LPS had no effects on PCV2 antigen localization. On the contrary, moDC displayed an accessory function, increasing cell proliferation and PCV2 replication in concanavalin A-stimulated PBMC. The latter effect required direct contact between moDC and PBMC [91]. 

PCV2 was able to infect blood cDC and pDC *in vitro*, without affecting their expression of MHC II and CD80/86. Interestingly, the virus compromised pDC cytokine responses to TLR-9 stimulation [92]. This pDC impairment was dependant on PCV2 DNA, but not viral replication [93]. In addition, circular viral DNA had a stronger inhibitory effect when compared to linear viral DNA [94]. This pDC inhibitory effect was not due to a reduction in CpG-ODN uptake, since PCV2-infected pDC efficiently internalized this TLR-9 ligand. In addition, the virus inhibited pDC IFN-β release in response to other stimuli: TLR-7 ligand (R837), TGEV, PRV, and CSFV [93]. This inability to activate pDC is in accordance with the low type I IFN levels that were found in the sera of PCV2 infected pigs [95], and this pDC impairment might also affect cDC maturation, with consequent impairment of the development of immune defence against PCV2 and other pathogens [92,93]. In addition, a link between PCV2 immunomodulatory capacity and cytoskeleton reorganization was observed: PCV2 viral DNA impaired both pDC actin polymerization and moDC endocytosis, which suggests an effect of this virus on DC danger recognition [93]. In 2013, Baumann et al. assessed which factors may increase pDC type I response to PCV2 and observed that, in the presence of IFN-γ, but not IFN-β, Flt3-L, GM-CSF, pDC were stimulated by this virus [96]. Interestingly, DNase treatment did not influence PCV-2 IFN-α release by IFN-γ activated pDC, suggesting that this stimulatory effect was mediated by the encapsulated viral ssDNA [96]. These results suggested a critical role of IFN-γ in sensing PCV2 by the innate immune system. 

In vivo, PCV2 antigen has been found in macrophages, DC, and follicular dendritic cells [97,98,99]. A recent study focused on moDC generated from blood monocytes of PCV-2 infected pigs [100]. It was observed that PCV2 infection in vivo impaired DC functions: at the peak of virus proliferation (7 days post-infection), moDC generated from infected pigs presented reduced MHC II and CD80/86 expressions, lower levels of IL-10, IL-12, IL-8, MIP-1β, and reduced ability to stimulate T cells. This DC dysfunction could be at least, in part, responsible for PCV2-induced immunosuppression [100]. Interactions of PCV2 with different DC subsets are summarized in Figure 5.

## 4. Conclusions

In the first part of this review we provided an update on porcine DC family. We hope to have conveyed the complexity of this system, which is composed of different subsets whose phenotype is partially organ-specific. When considering both the importance of pork as a source of animal protein for an expanding human population and the role of the pig as animal model in many fields of biomedical research, more studies are needed to better characterise the functional roles of DC subsets in this species. In the second part of this review, we summarized the main findings on three key porcine viruses’ modulation of DC phenotype and functionality. These viruses differently modulate DC functions: virulent ASFV and CSFV appear to have developed mechanisms to covertly replicate in myeloid DC, whereas PCV2 was able to persist in DC in the absence of virus replication or degradation, thus these viruses probably use these cells as a vehicle to spread through the host. PCV2 infection impaired pDC function, on the contrary, both ASFV and CSFV stimulated pDC, and this might be the source of the pathologically high type I IFN in animals undergoing these acute swine fevers. These studies on virus-DC interaction mainly focused on moDC and blood DC, and very few papers assessed virus interaction with skin, mucosal, tonsillar, or lymph node DC. In addition, differences in responses to infection between cDC subsets, derived from blood or from different tissue sites, were not investigated. We suggest that further studies in this area are needed, to better understand both diseases pathogenesis and mechanisms behind protection. Better knowledge regarding the immunobiology of porcine DC system and its interaction with pathogens will undoubtedly aid the rational design of improved vaccines. 

## References

[1] VanderWaal K., Deen J. (2018). Global trends in infectious diseases of swine. Proc. Natl. Acad. Sci. USA.

[2] Chambers M.A., Graham S.P., La Ragione R.M. (2016). Challenges in veterinary vaccine development and immunization. Methods Mol. Biol..

[3] Banchereau J., Steinman R.M. (1998). Dendritic cells and the control of immunity. Nature.

[4] Crisci E., Fraile L., Montoya M. (2019). Cellular innate immunity against PRRSV and Swine Influenza viruses. Vet. Sci..

[5] Medina G.N., Segundo F.D., Stenfeldt C., Arzt J., de Ios Santos T. (2018). The different tactics of foot-and-mouth disease virus to evade innate immunity. Front. Microbiol..

[6] Steinman R.M., Cohn Z.A. (1973). Identification of a novel cell type in peripheral lymphoid organs of mice. I. Morphology, quantitation, tissue distribution. J. Exp. Med..

[7] Kelsall B.L., Biron C.A., Sharma O., Kayle P.M. (2002). Dendritic cells at the host-pathogen interface. Nat. Immunol..

[8] Bancherau J., Briere F., Caux C., Davoust J., Lebecque S., Liu Y.J., Pulendran B., Palucka K. (2000). Immunobiology of dendritic cells. Annu. Rev. Immunol..

[9] Kleijmeer M.J., Ossevoort M.A., van Veen C.J., van Hellemond J.J., Neefjes J.J., Kast W.M., Melief C.J., Geuze H.J. (1995). MHC class II compartment and the kinetics of antigen presentation in activated mouse spleen dendritic cells. J. Immunol..

[10] Wykes M., Pombo A., Jenkins C., MacPherson G.G. (1998). Dendritic cells interact directly with naive B lymphocytes to transfer antigen and initiate class switching in a primary T-dependent response. J. Immunol..

[11] Trinchieri G. (2003). Interleukin-12 and the regulation of innate resistance and adaptive immunity. Nat. Rev. Immunol..

[12] Fehniger T., Caligiuri M. (2001). Interleukin 15: Biology and relevance to human disease. Blood.

[13] Gerosa F., Baldani-Guerra B., Nisii C., Marchesini V., Carra G., Trinchieri G. (2002). Reciprocal Activating Interaction between Natural Killer Cells and Dendritic Cells. J. Exp. Med..

[14] Devilder M., Allain S., Dousset C., Bonneville M., Scotet E. (2009). Early triggering of exclusive IFNγ responses of human γδT cells by TLR activated myeloid and plasmacytoid dendritic cells. J. Immunol..

[15] Collin M., Bigley V. (2018). Human dendritic cell subsets: An update. Immunology.

[16] Grouard G., Rissoan M., Filgueira L., Durand I., Banchereau J., Liu Y. (1997). The enigmatic plasmacytoid T cells develop into dendritic cells with interleukin (IL)-3 and CD40-Ligan. J. Exp Med..

[17] Liu Y. (2005). IPC: Professional type 1 interferin-producing cells and plasmacytoid dendritic cell precursors. Annu. Rev. Immunol..

[18] Granot T., Senda T., Carpenter D.J., Matsuoka N., Weiner J., Gordon C.L., Miron M., Kumar B., Griesemer A., Ho S.-H. (2017). Dendritic cells display subset and tissue-specific maturation dinamics over human life. Immunity.

[19] Summerfield A., McCullough K.C. (2009). The porcine dendritic cell family. Dev. Comp. Immunol..

[20] Summerfield A., Guzylack-Piriou L., Schaub A., Carrasco C.P., Tache V., Charley B., McCullough K.C. (2003). Porcine peripheral blood dendritic cells and natural interferon-producing cells. Immunology.

[21] Auray G., Keller I., Python S., Gerber M., Bruggmann R., Ruggli N., Summerfield A. (2016). Characterization and transcriptomic analysis of porcine blood conventional and plasmacytoid dendritic cells reveals striking species-specific differences. J. Immunol..

[22] Edwards J.C., Everett H.E., Pedrera M., Mokhtar H., Marchi E., Soldevila F., Kaven D.A., Hogarth P.J., Johns H.L., Nunez-Garcia J. (2017). CD1^−^ and CD1^+^ porcine blood dendritic cells are enriched for the orthologues of the two major mammalian conventional subsets. Sci. Rep..

[23] Vreman S., Auray G., Savelkoul H.F.J., Rebel A., Summerfield A., Stockhofe-Zurwieden N. (2019). Neonatal porcine blood derived dendritic cell subsets show activation after TLR2 or TLR9 stimulation. Dev. Comp. Immunol..

[24] Auray G., Facci M.R., Kessel J., Buchanan R., Babiuk L.A., Gerdts V. (2013). Porcine neonatal blood dendritic cells, but not monocytes, are more responsive to TLRs stimulation than their adult counterparts. PLoS ONE.

[25] Carrasco C.P., Rigden R.C., Schaffner R., Gerber H., Neuhaus V., Inumare S., Takamatsu H., Bretoni G., McCullough K.C., Summerfield A. (2001). Porcine dendritic cells generated in vitro: Morphological, phenotypic and functional properties. Immunology.

[26] Paillot R., Laval F., Audonnet J.C., Andreoni C., Juillard V. (2001). Functional and phenotypic characterization of distinct porcine dendritic cells derived from peripheral blood monocytes. Immunology.

[27] Chamorro S., Revilla C., Gomez N., Alvarez B., Alonso F., Ezquerra A., Dominguez J. (2004). In vitro differentiation of porcine blood CD163- and CD163+ monocytes into functional dendritic cells. Immunobiology.

[28] Bautista E.M., Nfon C., Ferman G.S., Golde W.T. (2007). IL-13 replaces IL-4 in development of monocyte derived dendritic cells (MoDC) of swine. Vet. Immunol. Immunopathol..

[29] Carrasco C.P., Rigden R.C., Vincent I.E., Balmelli C., Ceppi M., Bauhofer O., Tache V., Hjertner B., McNeilly F., van Gennip H.G. (2004). Interaction of classical swine fever virus with dendritic cells. J. Gen. Virol..

[30] Singleton H., Graham S.P., Bodman-Smith K.B., Frossard J.P., Steinbach F. (2016). Establishing porcine monocyte-derived macrophage and dendritc cell systems for studying the interaction with PRRSV-1. Front. Microbiol..

[31] Facci M.R., Auray G., Buchanan R., Kessel J., Thompson D.R., Mackenzie-Dyck S., Babiuk L.A., Gerdts V. (2009). A comparison between isolated blood dendritic cells and monocyte-derived dendritic cells in pigs. Immunology.

[32] Summerfield A., Meurens F., Ricklin M.E. (2015). The immunology of the porcine skin and its value as a model for human skin. Mol. Immunol..

[33] Schmook F.P., Meingassner J.G., Billich A. (2001). Comparison of human skin or epidermis models with human and animal skin in in-vitro percutaneous absorption. Int. J. Pharm..

[34] Marquet F., Vu Manh T., Maisonnasse P., Elhmouzi-Younes J., Urien C., Bouguyon E., Jouneau L., Bourge M., Simon G., Ezquerra A. (2014). Pig skin includes dendritic cell subsets transcriptomically related to human CD1a and CD14 dendritic cells presenting different migrating behaviours and T cell activation capacities. J. Immunol..

[35] Marquet F., Bonneau M., Pascale F., Urien C., Kang C., Schwartz-Cornil I., Bertho N. (2011). Characterization of dendritic cells subpopulations in skin and afferent lymph in the swine model. PLoS ONE.

[36] Nfon C.K., Dawson H., Toka F.N., Golde W.T. (2008). Langerhans cells in porcine skin. Vet. Immunol. Immunopathol..

[37] Jamin A., Gorin S., Le Potier M.F., Kuntz-Simon G. (2006). Characterization of conventional and plasmacytoid dendritic cells in swine secondary lymphoid organs and blood. Vet. Immunol. Immunopathol..

[38] Soldevila F., Edwards J.C., Graham S.P., Stevens L.M., Crudgington B., Crooke H.R., Werling D., Steinbach F. (2018). Characterization of the myeloid cell populations’ resident in the porcine palatine tonsil. Front. Immunol..

[39] Parra-Sanchez H., Puebla-Clark L., Resendiz M., Valenzuela O., Hernandez J. (2018). Characterization and expression of DEC205 in the cDC1 and cDC2 subsets of porcine dendritic cells from spleen, tonsil, and submaxillary and mesenteric lymph nodes. Mol. Immunol..

[40] Maisonnasse P., Bouguyon E., Piton G., Ezquerra A., Urien C., Deloizy C., Bourge M., Leplat J.J., Simon G., Chevalier C. (2016). The respiratory DC/macrophage network at steady-state and upon influenza infection in the swine biomedical model. Mucosal Immunol..

[41] Maisonnasse P., Bordet E., Bouguyon E., Bertho N. (2016). Broncho alveolar dendritic cells and macrophages are highly similar to their interstitial counterparts. PLoS ONE.

[42] Proll M.J., Neuhoff C., Schellander K., Uddin M.J., Cinar M.U., Sahadevan S., Qu X., Islam M.A., Poirier M., Muller M.A. (2017). Transcritome profile of lung dendritic cells after in vitro porcine reproductive and respiratory syndrome virus (PRRSV) infection. PLoS ONE.

[43] Tulman E.R., Delhon G.A., Ku B.K., Rock D.L. (2009). African swine fever virus. Curr. Top. Microbiol. Immunol..

[44] Blome S., Gabriel C., Beer M. (2013). Pathogenesis of African swine fever in domestic pigs and European wild boar. Virus Res..

[45] Arias M., de la Torre A., Dixon L., Gallardo C., Jori F., Laddomada A., Martins C., Parkhouse R.M., Revilla Y., Rodriguez F.M. (2017). Approaches and Perspectives for Development of African Swine Fever Virus Vaccines. Vaccines.

[46] OIE, WAHIS interface. https://www.oie.int/wahis_2/public/wahid.php/Wahidhome/Home.

[47] Sánchez-Cordón P.J., Romero-Trevejo J.L., Pedrera M., Sánchez-Vizcaíno J.M., Bautista M.J., Gómez-Villamandos J.C. (2008). Role of hepatic macrophages during the viral haemorrhagic fever induced by African swine fever virus. Histol. Histopathol..

[48] Franzoni G., Dei Giudici S., Oggiano A. (2018). Infection, modulation and responses of antigen-presenting cells to African swine fever viruses. Virus Res..

[49] Golding J., Goatley L., Goodbourn S., Dixon L., Taylor G., Netherton C. (2016). Sensitivity of African swine fever virus to type I interferon is linked to genes within multigene families 360 and 505. Virology.

[50] Franzoni G., Graham S.P., Sanna G., Angioi P., Fiori M., Anfossi G.A., Amadori M., Dei Giudici S., Oggiano A. (2018). Interaction of monocyte derived dendritic cells with African swine fever viruses of diverse virulence. Vet. Microbiol..

[51] Lanier L. (2005). NK cell recognition. Annu. Rev. Immunol..

[52] Leitão A., Cartaxeiro C., Coelho R., Cruz B., Parkhouse R.M., Portugal F., Vigário J.D., Martins C.L. (2001). The non-haemadsorbing African swine fever virus isolate ASFV/NH/P68 provides a model for defining the protective anti-virus immune response. J. Gen. Virol..

[53] Drummond R., Johnson L., Netherton C., Reis A., Morgan S., Graham S., Dixon L. Understanding the role of porcine dendritic cells in African swine fever infection. Proceedings of the 6th European Veterinary Immunology Workshop.

[54] Gregg A.D., Schlafer D.H., Mebus C.A. (1995). African swine fever virus infection of skin-derived dendritic cells in vitro causes interference with subsequent foot-and-mouth disease virus infection. J. Vet. Diagn. Investig..

[55] Gregg D.A., Mebus C.A., Schlafer D.H. (1995). Early infection of interdigitating dendritic cells in the pig lymph node with African swine fever viruses of high and low virulence: Immunohistochemical and ultrastructural studies. J. Vet. Diagn. Investig..

[56] Moennig V. (2000). Introduction to classical swine fever: Virus, disease and control policy. Vet. Microbiol..

[57] Thiel H., Stark R., Weiland E., Rumenapf T., Meyers G. (1991). Hog cholera virus: Molecular composition of virions from a pestivirus. J. Virol..

[58] Van Oirschot J.T. (2003). Vaccinology of classical swine fever: From lab to field. Vet. Microbiol..

[59] Moennig V. (2015). The control of classical swine fever in wild boar. Front. Microbiol..

[60] Simmonds P., Becher P., Collett M.S., Gould E.A., Heinz F.X., Meyers G., Monath T., Pletnev A., Rice C.M., Stiasny K., King A.M.Q., Adams M.J., Carstens E.B., Lefkowitz E.J. (2011). Family Flaviviridae. Virus Taxonomy: Ninth Report of the International Committee on Taxonomy of Viruses.

[61] Ruggli N., Bird B., Liu L., Bauhofer O., Tratschin J., Hofmann M. (2005). N(pro) of classical swine fever virus is an antagonist of double-stranded RNA-mediated apoptosis and IFN-α/β induction. Virology.

[62] La Rocca A., Herbert R., Crooke H., Drew T., Wileman T., Powell P. (2005). Loss of Interferon Regulatory Factor 3 in Cells Infected with Classical Swine Fever Virus Involves the N-Terminal Protease, Npro. J. Virol..

[63] Bauhofer O., Summerfield A., McCullough K.C., Ruggli N. (2005). Role of double-stranded RNA and Npro of classical swine fever virus in the activation of monocyte-derived dendritic cells. Virology.

[64] Husser L., Ruggli N., Summerfield A. (2012). Npro of classical swine fever prevents type I interferon mediated priming of conventional dendritic cells for enhanced interferon-α response. J. Interferon Cytokine Res..

[65] Chen L., Dong X., Shen H., Zhao M., Ju C., Yi L., Zhang X., Kang Y., Chen J. (2012). Classical swine fever virus suppress maturation and modulates functions of monocyte-derived dendritic cells without activating nuclear factor kappa B. Res. Vet. Sci..

[66] Santoro M.G., Rossi A., Amici C. (2003). NF-kappa B and virus infection: Who controls whom. EMBO.

[67] Jamin A., Gorin S., Cariolet R., Le Potier M., Kuntz-Simon G. (2008). Classical swine fever virus induces activation of plasmacytoid and conventional dendritic cells in tonsil, blood, and spleen of infected pigs. Vet. Res..

[68] Balmelli C., Vincent I.E., Rau H., Guzylack-Piriou L., McCullough K., Summerfield A. (2005). FcyRII-dependent sensitisation of natural interferon-producing cells for viral infection and interferon-α responses. Eur. J. Immunol..

[69] Fiebach A.R., Guzylack-Piriou L., Python S., Summerfield A., Ruggli N. (2011). Classical swine fever virus Npro limits type I interferon induction in plasmacytoid dendritic cells by interacting with interferon regulatory factor 7. J. Virol..

[70] Phyton S., Gerber M., Suter R., Ruggli N., Summerfield A. (2013). Efficient sensing of infected cells in absence of virus particles by plasmacytoid dendritic cells is blocked by the viral ribonuclease E(rns). PLoS Pathog..

[71] Franzoni G., Edwards J.C., Kurkure N.V., Edgar D., Sanchez-Cordon P., Haines F., Salguero F.J., Everett H.E., Bodman-Smith K.B., Crooke H.R. (2014). Partial activation of natural killer and γδ-T cells by classical swine fever virus does not correlate with the rapid protection conferred by live C-strain vaccination. Clin. Vaccine Immunol..

[72] Summerfield A., Alves M., Ruggli N., de Bruin M.G., McCullough K.C. (2006). High IFN-alpha responses associated with depletion of lymphocytes and natural IFN-producing cells during classical swine fever. J. Interferon Cytokine Res..

[73] Summerfield A., Ruggli N. (2015). Immune responses against classical swine fever virus: Between ignorance and lunacy. Front. Vet. Sci..

[74] Summerfield A., Knoetig S.M., McCullough K.C. (1998). Lymphocyte apoptosis during Classical Swine Fever: Implication of activation-induced cell death. J. Virol..

[75] Renson P., Blanchard Y., Le Dimna M., Felix H., Cariolet R., Jestin A., Le Potier M.F. (2010). Acute induction of cell death-related IFN stimulated genes (ISG) differentiates highly from moderately virulent CSFV strains. Vet. Res..

[76] Durand S.V., Hulst M.M., de Wit A.A., Mastebroek L., Loeffen W.F. (2009). Activation and modulation of antiviral and apoptotic genes in pigs infected with classical swine fever viruses of high, moderate or low virulence. Arch. Virol..

[77] Segalés J. (2012). Porcine circovirus type 2 (PCV2) infections: Clinical signs, pathology and laboratory diagnosis. Virus Res..

[78] Darwich L., Mateu E. (2012). Immunology of porcine circovirus type 2 (PCV2). Virus Res..

[79] Segalés J., Kekarainen T., Cortey M. (2013). The natural history of porcine circovirus type 2: From an inoffensive virus to a devasting swine disease?. Vet. Microbiol..

[80] Segalés J., Allan G.M., Domingo M. (2005). Porcine circovirus diseases. Anim. Health Res. Rev..

[81] Firth C., Charleston M.A., Duffy S., Shapiro B., Holmes E.C. (2009). Insights into the evolutionary history of an emerging livestock pathogen: Porcine Circovirus 2. J. Virol..

[82] Kekarainen T., Segales J. (2015). Porcine circovirus 2 immunology and viral evolution. Proc. Health Manag..

[83] McCullough K.C., Ruggli N., Summerfield A. (2009). Dendritic cells—At the front-line of pathogen attack. Vet. Immunol. Immunopathol..

[84] Kekarainen T., McCullough K., Fort M., Fossum C., Segales J., Allan G.M. (2010). Immune responses and vaccine-induced immunity against Porcine circovirus type 2. Vet. Immunol. Immunopathol..

[85] Vincent I.E., Carrasco C.P., Herrmann B., Meehan B.M., Allan G.M., Summerfield A., McCullough K.C. (2003). Dendritic cells harbor infectious porcine circovirus type 2 in the absence of apparent cell modulation or replication of the virus. J. Virol..

[86] Steiner E., Balmelli C., Herrmann B., Summerfield A., McCullough K. (2008). Porcine circovirus type 2 displays pluripotency in cell targeting. Virology.

[87] Yang N., Qiao J., Liu S., Zou Z., Zhu L., Liu X., Zhou S., Li H. (2017). Change in the immune function of porcine iliac artery endothelial cells infected with porcine cyrcovirus type 2 and its inhibition on monocyte derived dendritic cells maturation. PLoS ONE.

[88] Kekarainen T., Montoya M., Mateu E., Segales J. (2008). Porcine circovirus type 2-induced interleukin-10 modulates recall antigen responses. J. Gen. Virol..

[89] Kekarainen T., Montoya M., Dominguez J., Mateu E., Segales J. (2008). Porcine circovirus type 2 (PCV2) viral components immunomodulate recall antigen responses. Vet. Immunol. Immunopathol..

[90] Mavrommatis B., Offord V., Patterson R., Watson M., Kanellos T., Steinbach F., Grierson S., Werling D. (2014). Global gene expression profiling of myeloid immune cell subsets in response to in vitro challenge with porcine circovirus 2b. PLoS ONE.

[91] Lin C.M., Jeng C.R., Hsiaso S.H., Lee Y., Tsai Y.C., Chia M.Y., Pang V.E. (2012). Monocyte-derived dendritic cells enhance cell proliferation and porcine circovirus type 2 replication in concavalin A-stimulated swine peripheral blood lymphocytes in vitro. Vet. Immunol. Immunopathol..

[92] Vincent I.E., Carrasco C.P., Guzylack-Piriou L., Herrmann B., McNeilly F., Allan G.M., Summerfield A., McCullough K.C. (2005). Subset-dependent modulation of dendritic cell activity by circovirus type 2. Immunology.

[93] Vincent I.E., Balmelli C., Meehan B., Allan G., Summerfield A., McCollough K. (2007). Silencing of natural interferon producing cells activation by porcine circovirus type 2 DNA. Immunology.

[94] Balmelli C., Steiner E., Moulin H., Peduto N., Herrmann B., Summerfield A., McCullough K. (2011). Porcine circovirus type 2 DNA influences cytoskeleton rearrangements in plasmacytoid and monocyte-derived dendritic cells. Immunology.

[95] Forth M., Fernander L.T., Nofrarias M., Diaz I., Sibila M., Pujols J., Mateu E., Segales J. (2009). Development of cell-mediated immunity to porcine circovirus type 2 (PCV2) in casearean-derived, colostrum-deprived piglets. Vet. Immunol. Immunopathol..

[96] Baumann A., McCullough K.C., Summerfield A. (2013). Porcine circovirus type 2 stimulates plasmacytoid dendritic cells in the presence of IFN-gamma. Vet. Immunol. Immunopathol..

[97] Allan G.M., Ellis J.A. (2000). Porcine circoviruses: A review. J. Vet. Diagn. Investig..

[98] Krakowka S., Ellis J.A., McNeilly F., Gilpin D., Meehan B., McCullough K., Allan G. (2002). Immunologic features of porcine circovirus type 2 infection. Viral Immunol..

[99] Marruchella G., Valbonetti L., Bernabò N., Ligios C. (2017). Depletion of follicular dendritic cells in tonsils collected from PMWS-affected pigs. Arch. Virol..

[100] Yang N., Li J., Yang Q., Qiao J., Cui D., Liu F., Li H., Zhou S. (2018). Reduced antigen capability and modified inflammatory/immunosuppressive cytokine expression of induced monocyte-derived dendritic cells from peripheral blood of piglets infected with porcine circovirus type 2. Arch. Virol..

